# Differences in sleep difficulties between hospitalized patients with moderate dementia and people with preserved cognitive functions living in nursing homes

**DOI:** 10.1192/j.eurpsy.2023.1979

**Published:** 2023-07-19

**Authors:** I. Filipčić, K. Bosak, Ž. Bajić, V. Grošić

**Affiliations:** 1 Psychiatric Clinic Sveti Ivan, Zagreb; 2 Faculty of Dental Medicine and Health, Josip Juraj Strossmayer University of Osijek, Osijek; 3 School of Medicine, University of Zagreb, Zagreb, Croatia

## Abstract

**Introduction:**

Approximately 40% of people with dementia have some sleep disorder, and in as many as 20% this disorder is of clinically relevant severity. The risk of sleep disorders is especially high in people who have been hospitalized for a long time due to dementia. However, sleep disorders are also common in people hospitalized for a long time for the treatment of other mental disorders, so it is unclear to what extent the described risk for sleep disorders is related to dementia and to what extent to living in an institution. In order to answer that question, a control group of persons without dementia who are permanently housed in institutions is necessary. Given that the risk of sleep disorders is related to age and that people with dementia are older on average, a proper control population should be comparable in terms of age. The optimal control population therefore consists of persons without diagnosed dementia, i.e. preserved cognitive functions, permanently residing in homes for the elderly.

**Objectives:**

The aim of this research was to examine whether there are differences in sleep difficulties between hospitalized patients with moderate dementia and people with preserved cognitive functions living in homes for the elderly.

**Methods:**

Cross-sectional research at the Clinic for Psychiatry “Sveti Ivan”, Zagreb and five homes for the elderly in Zagreb. Sleep problems were measured using the Pittsburgh Sleep Quality Index. The hypothesis was tested using linear regression analysis with adjustment for age, sex, education and body mass index. The subjects were 60 patients diagnosed with moderate dementia, aged 60-90 years, who were treated for at least one month in a psychiatric hospital in the dementia department, and the control group was 60 people living in homes for the elderly.

**Results:**

The two groups were well matched in terms of age and sex, but there were large differences in the level of education and body mass index. After adjustment for the mentioned covariates, the total PSQI score was not statistically significantly different between the two groups (p = 0.839). The only statistically significant difference was that patients with dementia slept longer on average during the night (p = 0.003).

**Conclusions:**

Moderate dementia in hospitalized patients does not seem to be an independent risk factor for sleep difficulties.

**Disclosure of Interest:**

None Declared

